# Supplementary figures and images for: Herbivory induced methylation changes in the Lombardy poplar: A comparison of results obtained by epiGBS and WGBS
Source: PLoS One. 2023 Sep 8;18(9):e0291202. doi: 10.1371/journal.pone.0291202 (PMC10490839; doi:10.1371/journal.pone.0291202)

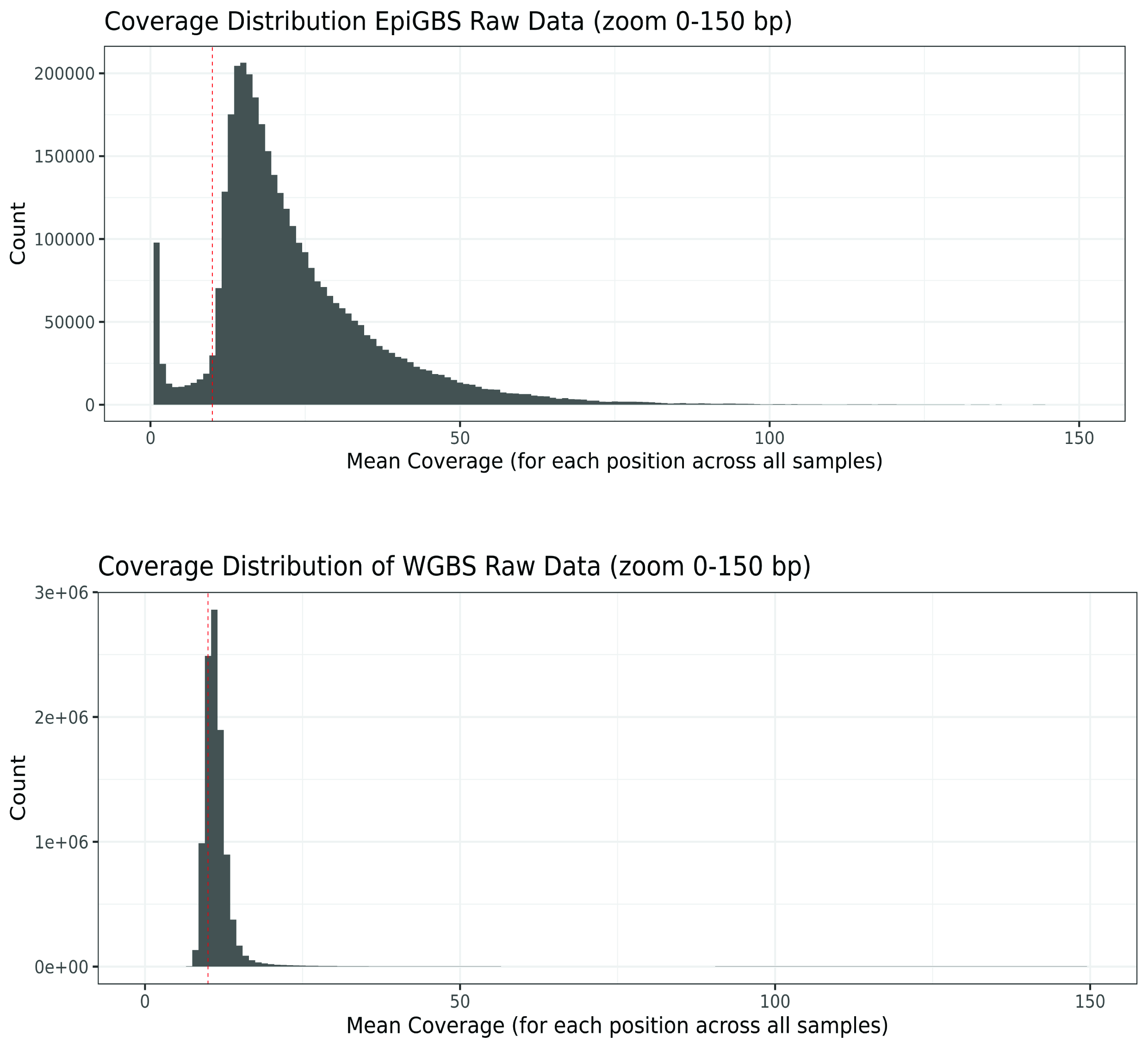

Supplement: S1 Fig — (TIF) [file pone.0291202.s001.tif]

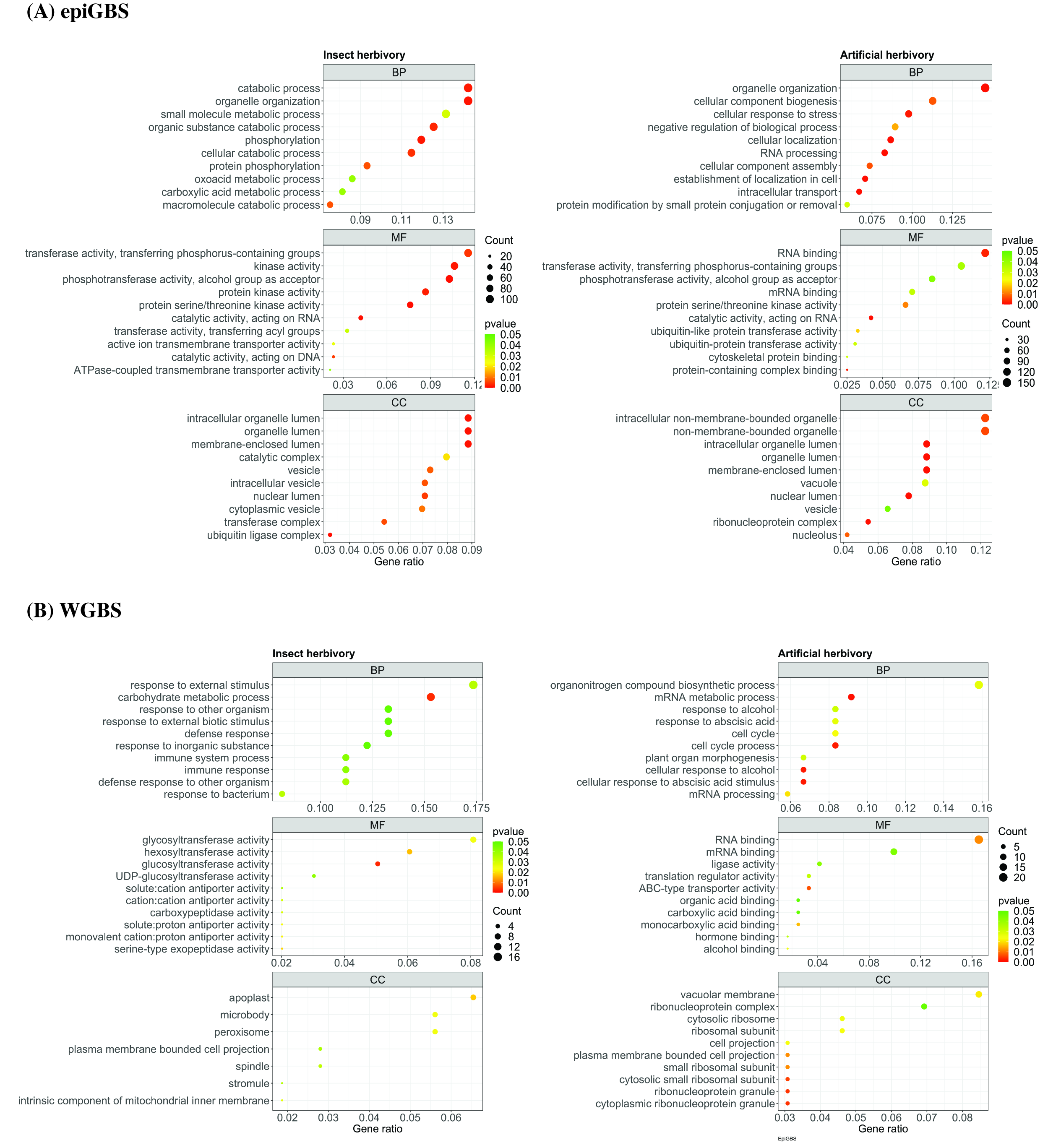

Supplement: S2 Fig — (TIF) [file pone.0291202.s002.tif]
