# Supplementary material for: Herbivory induced methylation changes in the Lombardy poplar: A comparison of results obtained by epiGBS and WGBS
Source: PLoS One. 2023 Sep 8;18(9):e0291202. doi: 10.1371/journal.pone.0291202 (PMC10490839; doi:10.1371/journal.pone.0291202)
Supplement: S1 Table — (DOCX) [file pone.0291202.s003.docx]

**S1 Table. Total number of differentially methylated cytosines for insect and artificial treated plants captured by WGBS and epiGBS-R (reference branch) in the three cytosine contexts.** In all the comparisons the filtering criteria was q-value<0.05 and methylation difference >10%.

| **Technique** | **Context** | **Insect** | | **Artificial** | |
| --- | --- | --- | --- | --- | --- |
|  |  | **#** | **%** | **#** | **%** |
| **epiGBS-R** | **CpG** | 1316 | 27.9 | 1822 | 30.6 |
|  | **CHG** | 1378 | 29.2 | 1991 | 33.4 |
|  | **CHH** | 2020 | 42.9 | 2148 | 36.0 |
| **WGBS** | **CpG** | 1346 | 69.0 | 1847 | 66.1 |
|  | **CHG** | 593 | 30.4 | 941 | 34.0 |
|  | **CHH** | 13 | 0.6 | 6 | 0.9 |
