# Supplementary material for: Herbivory induced methylation changes in the Lombardy poplar: A comparison of results obtained by epiGBS and WGBS
Source: PLoS One. 2023 Sep 8;18(9):e0291202. doi: 10.1371/journal.pone.0291202 (PMC10490839; doi:10.1371/journal.pone.0291202)
Supplement: S2 Table — (DOCX) [file pone.0291202.s004.docx]

**S2 Table. Total number of differentially methylated cytosines (DMC) captured by epiGBS-R (reference branch) and WGBS in the three sequence contexts (CpG, CHG and CHH) in each genomic feature (promoter, down-stream of transcriptional sites, gene body and intergenic region), within transposable elements (TE) or outside (no TE) also indicated.** DMCs were defined by a minimum coverage of 10X for epiGBS and 6X for WGBS, 10 % change in methylation percentage and q-value <0.05. Higher and lower methylation shifts after herbivory relative to the methylation status in controls are represented respectively as hyper-methylated and hypo-methylated DMCs.

| **Technique** | **Context** | **Herbivory** | **Genomic feature** | **TE** | | **no TE** | |
| --- | --- | --- | --- | --- | --- | --- | --- |
|  |  |  |  | **hyper** | **hypo** | **hyper** | **hypo** |
| epiGBS-R | CpG | insect | promoter | 22 | 27 | 79 | 90 |
|  |  |  | down | 46 | 33 | 85 | 80 |
|  |  |  | gene body | 20 | 22 | 164 | 171 |
|  |  |  | intergenic | 87 | 81 | 169 | 140 |
|  |  | artificial | promoter | 32 | 70 | 84 | 120 |
|  |  |  | down | 52 | 39 | 115 | 122 |
|  |  |  | gene body | 24 | 29 | 219 | 259 |
|  |  |  | intergenic | 104 | 128 | 156 | 269 |
|  | CHG | insect | promoter | 44 | 22 | 66 | 61 |
|  |  |  | down | 56 | 29 | 65 | 42 |
|  |  |  | gene body | 45 | 32 | 189 | 171 |
|  |  |  | intergenic | 181 | 104 | 169 | 102 |
|  |  | artificial | promoter | 73 | 56 | 90 | 59 |
|  |  |  | down | 83 | 36 | 56 | 60 |
|  |  |  | gene body | 58 | 44 | 241 | 289 |
|  |  |  | intergenic | 249 | 192 | 207 | 198 |
|  | CHH | insect | promoter | 100 | 24 | 84 | 34 |
|  |  |  | down | 92 | 24 | 76 | 28 |
|  |  |  | gene body | 77 | 17 | 97 | 36 |
|  |  |  | intergenic | 633 | 222 | 367 | 109 |
|  |  | artificial | promoter | 123 | 37 | 84 | 38 |
|  |  |  | down | 88 | 45 | 70 | 31 |
|  |  |  | gene body | 76 | 30 | 92 | 53 |
|  |  |  | intergenic | 709 | 185 | 386 | 101 |
| WGBS | CpG | insect | promoter | 21 | 23 | 44 | 45 |
|  |  |  | down | 50 | 20 | 52 | 70 |
|  |  |  | gene body | 20 | 23 | 245 | 380 |
|  |  |  | intergenic | 112 | 59 | 90 | 92 |
|  |  | artificial | promoter | 17 | 50 | 60 | 103 |
|  |  |  | down | 24 | 54 | 58 | 147 |
|  |  |  | gene body | 16 | 45 | 334 | 384 |
|  |  |  | intergenic | 162 | 114 | 90 | 189 |
|  | CHG | insect | promoter | 8 | 18 | 7 | 9 |
|  |  |  | down | 11 | 5 | 11 | 13 |
|  |  |  | gene body | 13 | 11 | 94 | 236 |
|  |  |  | intergenic | 46 | 35 | 38 | 38 |
|  |  | artificial | promoter | 6 | 17 | 16 | 25 |
|  |  |  | down | 7 | 19 | 9 | 32 |
|  |  |  | gene body | 15 | 31 | 202 | 333 |
|  |  |  | intergenic | 98 | 41 | 32 | 58 |
|  | CHH | insect | promoter | 2 | 2 | 2 | 1 |
|  |  |  | intergenic | 3 | 3 | - | - |
|  |  | artificial | intergenic | 6 | - |  |  |
