# Supplementary material for: Herbivory induced methylation changes in the Lombardy poplar: A comparison of results obtained by epiGBS and WGBS
Source: PLoS One. 2023 Sep 8;18(9):e0291202. doi: 10.1371/journal.pone.0291202 (PMC10490839; doi:10.1371/journal.pone.0291202)
Supplement: S1 Appendix — (PDF) [file pone.0291202.s006.pdf]

Appendix A

Technical analysis of global methylation with fragments shared by WGBS and epiGBS

Methods

We searched for the common fragments obtained in epiGBS-R and WGBS (Table A1). Only those fragments with substantial methylation information: ≥ 5 cytosines for CpG or CHG, and ≥10 cytosines for CHH were retained and their average methylation level calculated. Linear models were applied to fragment methylation data to test for the effects of herbivory treatment (with three levels), ortet (with three levels), and technique (epiGBS-R vs WGBS) as fixed factors. Significance of fixed factors and their interaction was tested using function ANOVA (package car, v3.0-12.)[1]. The frequency of the average methylation percentage of shared fragments for each herbivory among ortets was compared between two methods by Pearson's Chi-square test, using chisq.test function of stats R package v3.6.2).

Finally, principal components analyses were run to detect multivariate patterns of DNA methylation variation among ramets assigned to different herbivory treatments and the effect of technique applied. The analysis was conducted on average methylation of the shared fragments obtained by epiGBS-R and WGBS, using the correlation matrix and the built-in R function "prcomp" of the stats package with default parameters.

Table A1. Total number of common methylated positions captured by epiGBS-R (reference branch) and WGBS in the three sequence contexts (CpG, CHG and CHH) with and without missing values (With NAs and Without NAs). Values were obtained using methylkit and taking into account positions with methylation information of ≥5 cytosines for CpG or CHG, and ≥10 cytosines for CHH in WGBS; and ≥10 cytosines for all contexts in epiGBS-R.

Technique	Context	With NAs	Without NAs	
		#	#	%
epiGBS-R	CpG	28,826	2,243	7.78
	CHG	30,954	3,771	12.18
	CHH	35,375	6,781	19.17
WGBS	CpG	32,459	1,569	4.83
	CHG	35,209	3,246	9.22
	CHH	38,907	7,222	18.56

Results

For the subset of shared fragments, the estimates of global cytosine methylation level in the leaf genomes of the poplar ramets studied ranged from 3.9% to 26.1% according to WGBS and from 6.1% to 24.2% according epiGBS-R. The ANOVA tests detected that methylation levels obtained by the two techniques were significantly different in the three contexts (A1 Fig; $P < 0.005$) for these common fragments. Average methylation was relatively higher in WGBS for CpG and CHG, whereas CHH methylation percentage was higher in epiGBS-R (A1 Fig), differences being statistically significant only in CHG and CHH contexts (Table A2). Furthermore, global methylation in leaf genomes did not differ between ortets, or the three herbivory levels, or the interaction between the study factors was never statistically different but were always significantly different in the three cytosine sequence contexts between the techniques (Table A2).

Table A2. Summary of the ANOVA results carried out to test the effect of ortet, herbivory treatment, technique and their interaction on genome wide DNA methylation for each of the three cytosine contexts performed with the common fragments of epiGBS-R and WGBS techniques.

**Context**	**Source of variation**	**d.f.**	**F**	**p**
CpG	ortet	2	0.03	0.97
	herbivory	2	0.53	0.61
	technique	1	107.36	**<0.0001**
	ortet × herbivory	4	0.67	0.61
	ortet ×technique	2	0.51	0.60
	ortet × herbivory × technique	4	0.84	0.50
CHG	ortet	2	0.34	0.71
	herbivory	2	0.35	0.70
	technique	1	185.08	**<0.0001**
	ortet × herbivory	4	0.61	0.65
	ortet × technique	2	0.16	0.85
	ortet × herbivory × technique	4	0.82	0.52
CHH	ortet	2	0.78	0.46
	herbivory	2	0.19	0.82
	technique	1	117.63	**<0.0001**
	ortet × herbivory	4	0.56	0.56
	ortet × technique	2	0.90	0.89
	ortet × herbivory × technique	4	0.69	0.69

Heatmap representation (A2 Fig) of average fragment methylation showed congruency between the two techniques for all three contexts across all 27 ramets (x-axis) and scaffolds (y-axis) and a large difference in the patterns observed for each context. Fragments had frequently very high or very low methylation in CpG and CHG contexts, with a lack of cases with intermediate methylation in those two contexts, whereas in CHH average methylation per fragment varies between low and intermediate but almost never reach a methylation > 50 %.

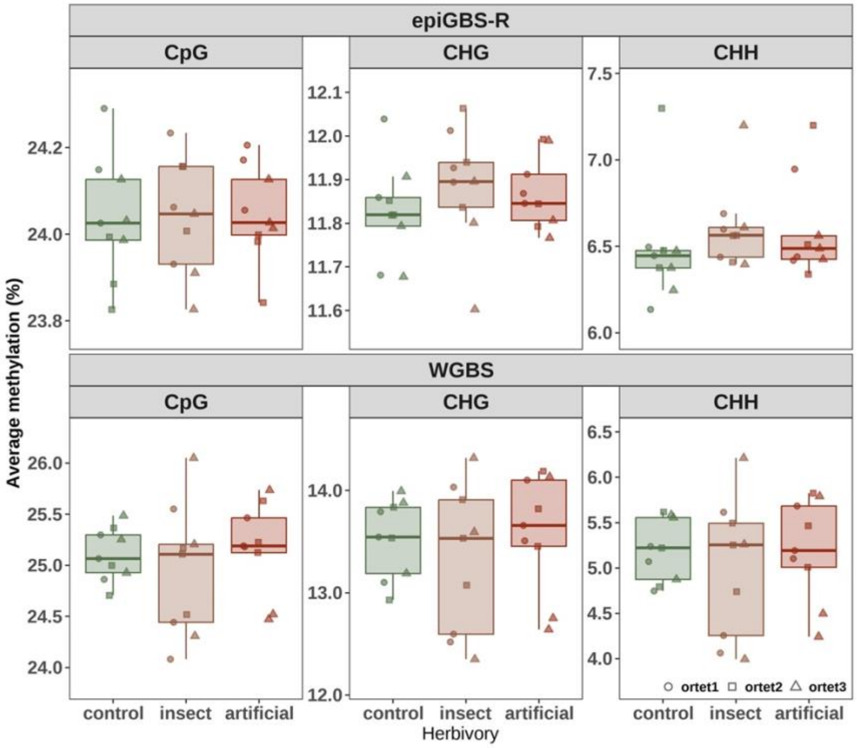

A1 Fig: Average methylation percentage estimated from common fragments captured by epiGBS-R and WGBS. (a) Boxplots of the average global DNA methylation level (%) obtained for controls, insect and artificial herbivory treated plants in each context (CpG, CHG and CHH) obtained by epiGBS-R and WGBS techniques. Treatments are shown in different colors (control: green, insect herbivory: brown, and artificial herbivory: red).

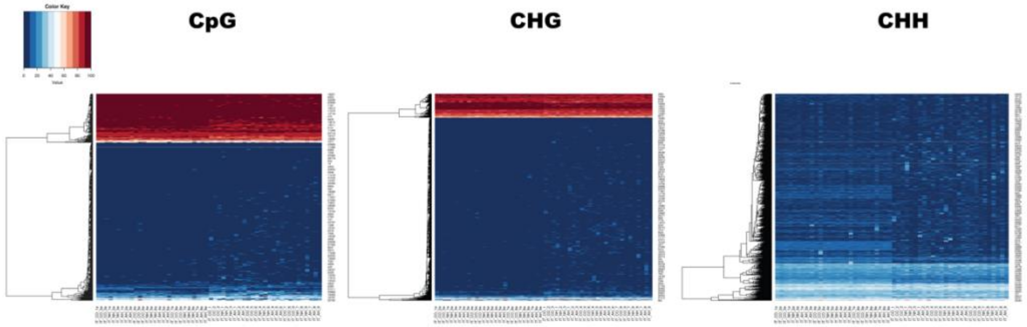

A2 Fig: Heatmap visualization of per-fragment average methylation based on common fragments in the three sequence contexts for each of the 27 samples that are shown ordered in the x-axis (left: epiGBS-R, right: WGBS). Hierarchical clustering (Ward's method) was performed on the fragments' methylation estimate (Manhattan distance). A scale is shown on the right, in which blue and red correspond to a lower and a higher methylation status, respectively.

As expected, principal component analysis showed that the primary source of variation in average methylation per fragment (PC1) was associated with the technique applied in all three contexts (29.8%, 30% and 21.1% for CG, CHG and CHH, respectively). Samples from each technique clustered together more closely along the PC1 principal component (A3 Fig). The second and third components (PC2 and PC3) explained only between 3.9% and 8.0 % of the variance in average methylation per fragment for the three different contexts. In CpG and CHG contexts, samples from each of the three study ortets clustered separately along the PC2 and PC3 axes, indicating that fragment methylation patterns differ between ortets, with ortet1 being the most distinct in both CpG and CHG contexts. However, in CHH context, PC2 and PC3 did not reveal any differences between ortets, treatments or techniques, and a larger variation among WGBS samples was observed when compared to variation among epiGBS-R samples.

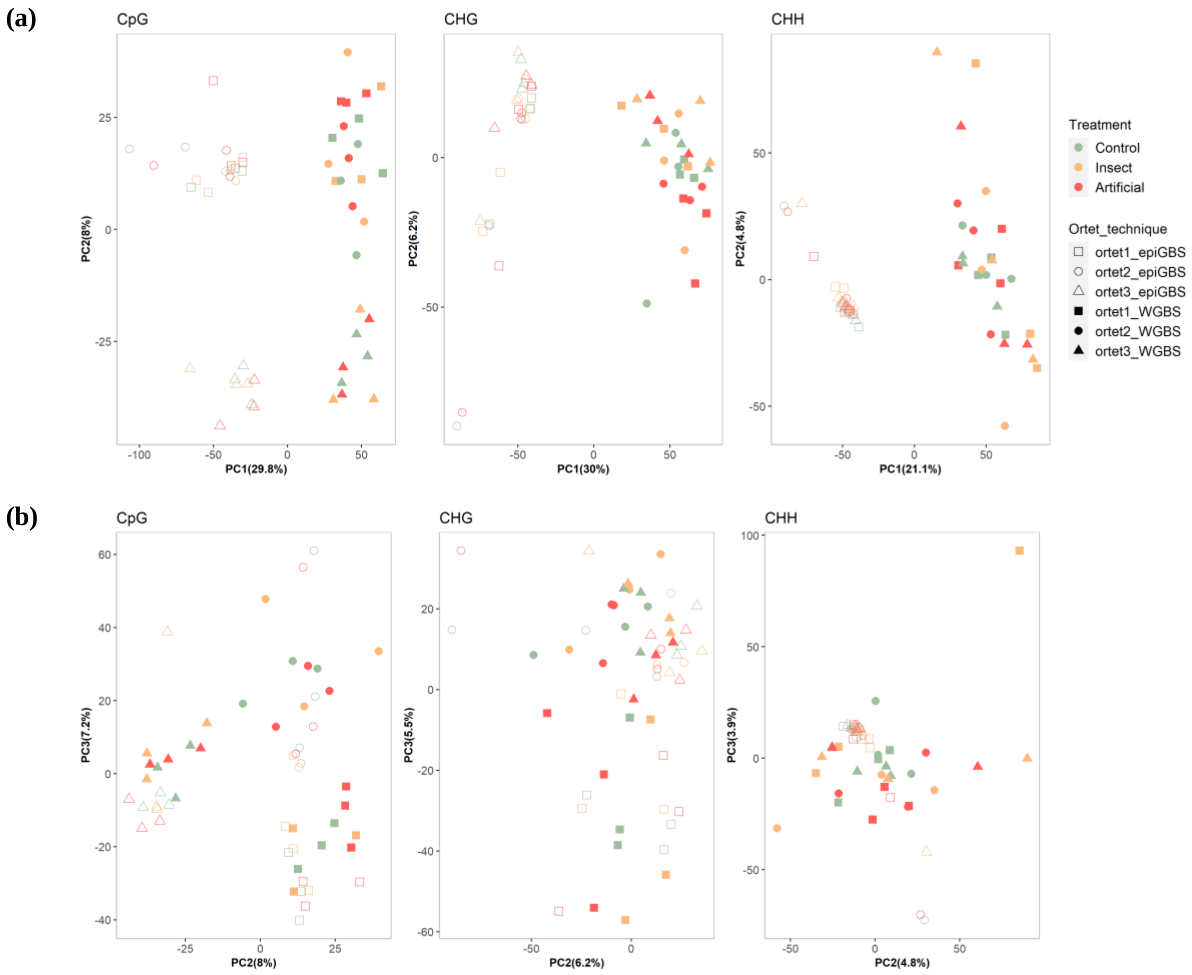

A3 Fig: PCA ordination plots on the DNA methylation profiles of the common loci captured by both epiGBS-R and WGBS in CpG, CHG and CHH context. (a) PC1 versus PC2. (b) PC2 versus PC3. Ortets are shown in different shapes and herbivory treatments are represented with different colors (control: green, insect: yellow, artificial: red). Filled and empty shapes represent WGBS and epiGBS samples, respectively.

1. J. Fox, S. Weisberg. An R companion to applied regression. 2019;
