# Supplementary material for: Herbivory induced methylation changes in the Lombardy poplar: A comparison of results obtained by epiGBS and WGBS
Source: PLoS One. 2023 Sep 8;18(9):e0291202. doi: 10.1371/journal.pone.0291202 (PMC10490839; doi:10.1371/journal.pone.0291202)
Supplement: Attachment — Submitted filename: Comments.docx [file pone.0291202.s007.docx]

Both EpiGBS and WGBS utilize bisulfite treatment and sequencing to study DNA methylation, EpiGBS is a targeted approach focusing on specific genomic regions, whereas WGBS provides a comprehensive analysis of the entire genome. The choice between these techniques depends on the research goals, available resources, and the desired level of coverage and resolution.

The study is good and well designed. I have few questions and comments:

Comment:

Poor formatting, figure legends look misplaced. The legends look like the part of results and have no sense where they are placed. It makes this article difficult to understand.

Questions:

Fig S1

WGBS covers the whole genome which contains repetitive region while epiGBS covers specific genomic regions, so the results are expected.

Table 1

Name NA is confusing, do the authors have any specific reason to use name with/without NA? I am not able to connect it with study design.

In Table 1 epiGBS-R/D captured most cytosines in CHH condition while WGBS has least %age cytosine capture in CHH. What is your view on this?

Table 2

In epiGBS-R/D, ortet shows significant methylation at CpG, herbivory show significant methylation at CHH. Ortet and herbivory interaction has no significant methylation. What is the biological meaning of this result?

Table S1

Why does WGBS have less DMCs than epiGBS-R (line 326)?

Overall, the study says that epiGBS offers reliable methylation insight. But the major drawback is that WGBS is performing better than epiGBS in functional analysis, so how epiGBS can be useful for actual biological system?
